# Malaria and Malarial Diseases

**Published:** 1884-10

**Authors:** 


### Book Reviews.

---

Malaria and Malarial Diseases. By George M. Sternberg, M.D., F.R.S., Major and Surgeon in the United States Army; member Biological Society of Washington; late member Havana Yellow Fever Commission of the National Board of Health; Corresponding member of the Epidemiological Society of London, etc. Wm. Wood & Co., New York, 1884.

This book, from the pen of Dr. Sternberg, comes to us as the July number of Wood's Library of Standard Medical Authors. We congratulate the publishers and felicitate the profession upon its appearance.

The subject is well chosen and practical, the author able, experienced, scientific and pains-taking. The publishers have spared no pains to execute his work in first class style. We regard it as equal to any of the series of valuable and cheap literature that the profession are indebted to this house for in the past.

Dr. Sternberg has exhausted the subject; is perfectly impartial, and has added to his large experience in the treatment and observations of malarial diseases, scientific observation—which granting others have—few possess the industry that this book bears testimony to.

We know of no book that should be more generally read than this, on account of the almost universal prevalence of these diseases, and wish for the edification of the profession and good of their patients that every physician in the South had a copy.

